# Prioritising Appointments by Telephone Interview: Duration from Symptom Onset to Appointment Request Predicts Likelihood of Inflammatory Rheumatic Disease

**DOI:** 10.3390/jcm13154551

**Published:** 2024-08-04

**Authors:** Martin Feuchtenberger, Magdolna Szilvia Kovacs, Axel Nigg, Arne Schäfer

**Affiliations:** 1 MVZ MED BAYERN OST, Rheumatologie, 84489 Burghausen, Germany; magdolna.kovacs@med-bayern-ost.de (M.S.K.); axel.nigg@innklinikum.de (A.N.); 2 University Hospital Würzburg, Medizinische Klinik und Poliklinik II, 97080 Würzburg, Germany; arne.schaefer@me.com; 3 Diabetes Zentrum Mergentheim, 97980 Bad Mergentheim, Germany

**Keywords:** musculoskeletal diseases, rheumatic diseases, early arthritis clinic, triage, appointments, prioritisation

## Abstract

**Background:** This study aims to determine the rate of inflammatory rheumatic diseases (IRDs) in a cohort of initial referrals and the efficacy of prioritising appointments to the early arthritis clinic (EAC) based on symptom duration. **Methods**: In the present study, we used algorithm-based telephone triage to assign routine care appointments according to the time between symptom onset and request for an appointment (cut-off criterion: 6 months). This retrospective, monocentric analysis evaluated the effectiveness of our triage in identifying patients with IRDs as a function of the assigned appointment category (elective, EAC, or emergency appointment). **Results**: A total of 1407 patients were included in the study (34.7% male; 65.3% female). Of the 1407 patients evaluated, 361 (25.7%) presented with IRD. There were significant differences in the frequency of inflammatory diagnoses between appointment categories (*p* < 0.001): elective 13.8%, EAC 32.9%, and emergency 45.9%. The sample without the emergency category included a total of 1222 patients. The classification into “inflammatory” or “non-inflammatory” in this subsample was as follows: Sensitivity was 37.7%, and specificity was 92.6%. The positive predictive value (PPV) was 59.8%, and the negative predictive value (NPV) was 83.6%. Overall, 80.2% of patients were correctly assigned using the appointment category and C-reactive protein (CRP). **Conclusions**: The algorithm-based triage system presented here, which focuses on the time between symptom onset and request for an appointment, allows for the prioritisation of appointments in favour of patients with IRDs and thus earlier initiation of therapy.

## 1. Introduction

Early diagnosis and initiation of specific therapy in inflammatory rheumatic diseases (IRDs) are of high prognostic importance [1]. Therefore, scientific guidelines worldwide consistently emphasise the goals of early diagnosis and initiation of specific therapy to achieve remission in IRDs [2]. Structural and thus irreversible damage to joints or organs and the resulting functional deficits can be reduced or prevented by the initiation of disease-modifying antirheumatic drugs (DMARDs) [2]. However, timely rheumatologic evaluation often fails due to the limited number of outpatient and inpatient rheumatology appointments [3]. This situation will worsen in the foreseeable future due to demographic trends in Western industrialised countries, such as Germany; due to ageing, the number of patients to be treated will increase, while the number of practising rheumatologists will decrease [3]. This is exacerbated by the high referral rate of patients with non-inflammatory conditions, such as degenerative diseases or primary pain disorders. Against this background, there is a need for efficient screening methods: The goal must be to prioritise access to specialised rheumatological care for patients with a high likelihood of an IRD, thereby ensuring they benefit from early diagnosis and specific therapy. Various approaches already exist, such as mobile screening units, run-in consultations with only orienting triage of the patient on site, online-based symptom checkers or portals, or just viewing submitted reports [4,5,6,7,8]. The objectives and requirements for such screening vary greatly depending on the regional conditions of rheumatologic care as well as the care mandate of the institution [7]. In this respect, there will not be one approach that is equally suitable for every region and every institution. The approach presented in this paper is aimed at primary care, outpatient settings, and is based on a structured interview conducted during the appointment request by patients for an initial rheumatology work-up. Based on the assessment of the likelihood of the presence of IRD, the urgency of the patient’s appointment is determined.

## 2. Materials and Methods

### 2.1. Patient Recruitment and Data Collection

**Figure 1:**
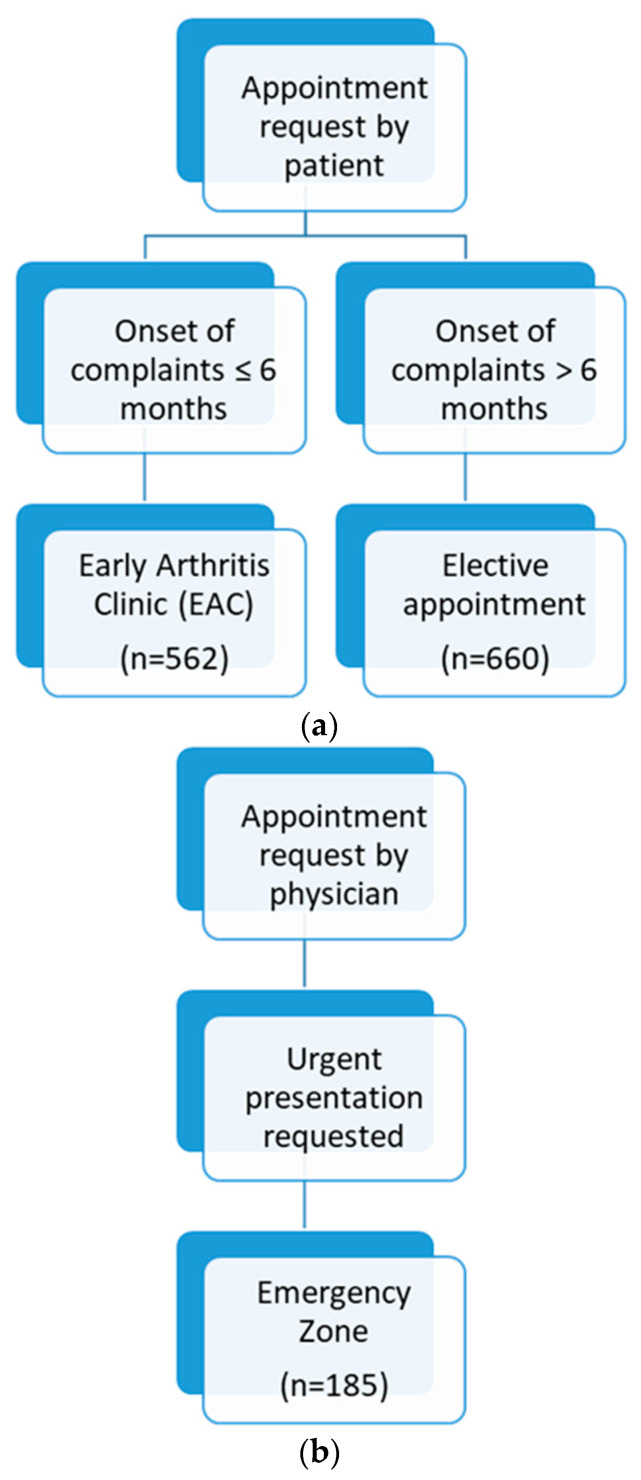
Overview of triage algorithm: appointment request by patient (**a**) and physician (**b**).

For the present study, we retrospectively analysed the digital patient records of a total of 1407 patients in our rheumatology outpatient clinic who underwent our telephone-based triage. None of the patients had a history of IRD or had been evaluated by a rheumatologist in the past. All patients had previously been seen by a physician, usually a general practitioner or orthopaedic specialist, and were referred to our department for rheumatological assessment. We investigated the feasibility of using a single telephone enquiry to determine the urgency of the patient’s appointment and to assign patients to one of the following appointment categories: “elective appointment”, “early arthritis clinic” (EAC), or “emergency appointment”. If the referred patient called for an appointment, our telephone triage only asked for the duration of symptoms, and an appointment was allocated based on the respective answer: if the duration of symptoms was >6 months, an “elective appointment” was given, and if ≤6 months, an “early arthritis clinic” (EAC) appointment was given. The last of these appointment categories (“emergency appointment”) was only given if the referring doctor called and asked for an urgent appointment. Patients who initially contacted us online (via email) or through a referral letter were called back, and the telephone interview was conducted. The respective flow chart can be seen in Figure 1. The total sample of the considered data sets originates from the time interval between 1 January 2018, and 15 December 2018. Elective appointments had a waiting period of 12–16 weeks, EAC had a waiting period of 4–6 weeks, and emergency appointments had a waiting period of no more than 2 weeks. All patients were seen by a rheumatologist and were diagnosed according to one of the following categories: non-inflammatory (e.g., osteoarthritis, fibromyalgia), rheumatoid arthritis (RA), axial spondyloarthritis, peripheral spondyloarthritis, other forms of arthritis (e.g., arthritis urica, Lofgren’s disease, arthritis urica), other forms of inflammation (e.g., polymyalgia rheumatica, autoinflammatory syndromes), connective tissue diseases, and vasculitis. We determined the erythrocyte sedimentation rate (ESR) by the Westergren method (normal value 6–11 mm during the first hour), and we measured highly sensitive C-reactive protein (CRP) levels by particle-enhanced immunonephelometry (F. Hoffmann-La Roche Diagnostics; normal value < 0.5 mg/dL). All data were analysed in the context of routine care according to available recommendations and guidelines. No additional inclusion or exclusion criteria were applied.

### 2.2. Statistical Analyses

The gained data were managed and statistically analysed using Microsoft Excel (German version 16.87) and/or SPSS (SPSS for Windows Release 17.0) [9], whichever was more appropriate for the respective purpose. Microsoft Excel was also used to create tables and graphs. All inferential tests were two-tailed and considered statistically significant at a probability level of *p* < 0.05. To compare the frequencies of categorical variables between patient subgroups, we utilised Pearson chi-square tests. One-way analysis of variance (ANOVA) was used to examine mean differences in continuous variables between independent patient subgroups. For statistical analyses of prediction questions, we utilised binary logistic regression procedures (“enter” option; dependent variable: observed dichotomised diagnosis; independent variables: e.g., laboratory data, results of the telephone interview). The results of the binary logistic regression analyses are presented in classification tables along with additional summary statistics (e.g., positive predictive value (PPV), negative predictive value (NPV), percentage of correctly classified cases—of course, together with indication of the associated *p* value). The study was conducted in accordance with the Declaration of Helsinki and approved by the Institutional Review Board of Würzburg University (# 207/21-me on 13 July 2021).

## 3. Results

A total of 1407 patients were included in the present study (34.7% men [n = 488], 65.3% women [n = 919]). An overview of the sociodemographic and medical characteristics of the included study sample is presented in Table 1.

**Figure 2:**
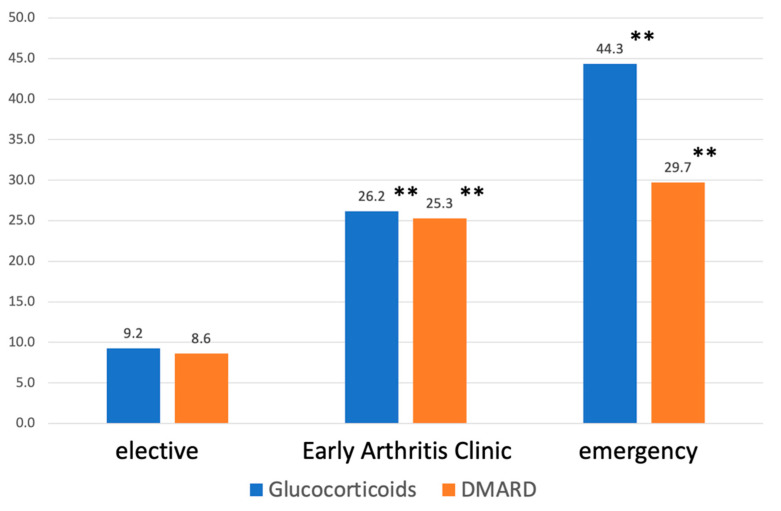
Percentage of patients with (initiation of) specific medication by appointment category. The *p* values shown (**, *p* < 0.001) refer to a higher proportion of specific medication (applies to glucocorticoids and to DMARDs) in the early arthritis clinic or emergency group in direct comparison with the elective group.

Of the 1407 patients evaluated, 361 (25.7%) were finally diagnosed with IRD. In the complementary patient group, inflammatory rheumatic disease was excluded in 1046 patients (74.3%). There were significant differences in the frequency of inflammation between appointment categories (*p* < 0.001): elective 13.8% [91 of 660], EAC 32.9% [185 of 562], and emergency 45.9% [85 of 185]. Post hoc analyses showed that all three pairwise comparisons between the appointment categories also showed statistically significant differences with regard to the prevalence of inflammatory rheumatic diseases (pairwise chi-square tests with corresponding alpha adjustment [Bonferroni correction]). Accordingly, the proportion of patients initiating therapy with glucocorticoids (9.2% [61 of 660]) or DMARDs (8.6% [57 of 660]) was very low in the elective appointment category. Significantly more therapy initiations were found in the appointment categories EAC and emergency zone (*p* < 0.001 for both DMARD and glucocorticoid therapy initiations). Comparable proportions of patients starting DMARD therapy were seen (29.7% [55 of 185] emergency zone vs. 25.3% [142 of 562] EAC), and the clearly highest incidence of new glucocorticoid therapy was seen in the emergency group (44.3% [82 of 185] emergency zone vs. 26.2% [147 of 562] EAC). The respective results are summarised and displayed in Figure 2.

**Figure 3:**
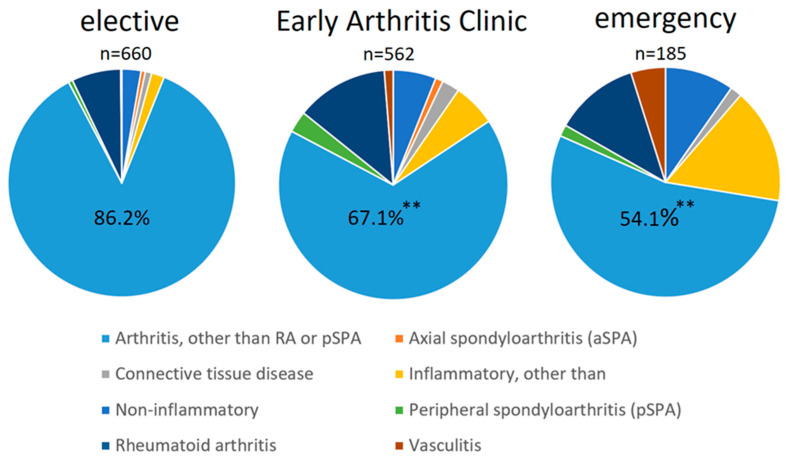
Distribution of diagnoses (inflammatory vs. non-inflammatory) stratified for appointment category (**, *p* < 0.001). The group “Arthritis, other than RA or pSPA” includes patients with inflammatory joint diseases such as gouty arthritis, reactive arthritis, or infectious arthritis (e.g., bacterial).

We wanted to examine in the total sample (n = 1407) how well the categorisation based on the presented triage is able to predict IRDs. The distribution of diagnoses (non-inflammatory vs. inflammatory with itemised specific diagnoses) is shown in Figure 3, separately for the three appointment categories. Performing a binary logistic regression analysis (dependent variable: inflammatory rheumatic disease, yes vs. no), we were able to show that the algorithm used (see Figure 1) correctly assigned a total of 79.1% (1113 of 1407) of the patients (sensitivity 45.7% [165 of 361 patients]; specificity 90.6% [948 of 1046 patients]). These classification results were based on the inclusion of two independent predictor candidates, namely appointment category (Exp(B) = 0.475; *p* < 0.001) and CRP (Exp(B) = 5.549; *p* < 0.001). Thus, this tested the scenario of how additionally collected inflammatory markers (here, CRP) could improve prediction. The interview-based triage without CRP correctly assigned 73.3% of patients [1031 of 1407]. In a second step, we wanted to check the predictive quality and value of our appointment allocation excluding the emergency category: how well does the triage process work for the non-emergency appointments (elective appointment and EAC)? The subsample of patients without the emergency category included a total of 1222 patients. In this subgroup, we also used binary logistic regression analysis to test the ability of the allocation algorithm in combination with CRP to detect or predict inflammatory rheumatic disease as a screening tool. The classification into “inflammatory” or “non-inflammatory” in this subsample was as follows: Sensitivity was 37.7% [104 of 276 patients], and specificity was 92.6% [876 of 946 patients]. The PPV was 59.8% [104 of 174 patients], and the NPV was 83.6% [876 of 1048 patients]. Overall, 80.2% of patients [980 of 1222] were correctly assigned using this approach. The prediction model was statistically significant with the independent variables in the model having the following characteristics: appointment category (Exp(B) = 0.348; *p* < 0.001) and CRP (Exp(B) = 5.464; *p* < 0.001). Here, the correspondent interview-based triage without CRP correctly assigned 77.4% of patients [946 of 1222].

## 4. Discussion

The introduction of new diagnostic tools and new agents, as well as new therapeutic strategies, has significantly contributed to the improvement in the prognosis of patients with IRDs in recent decades [10,11]. Of particular importance is the principle of early diagnosis followed by immediate initiation of specific therapy afterwards [12]. This can not only prevent irreversible, structural damage to joints and organs but can also favourably influence the course of the disease and reduce the risk of sequelae, such as infections or malignancies, as well as cardiovascular mortality [13]. However, all this progress is offset by a shortage of rheumatology appointments. Delays in the access process to rheumatologic care are considered a significant barrier and have been shown to occur at three main points: the time between symptom onset and contact with the primary care physician, the time to referral to a specialist, and, finally, the waiting time to be seen by a rheumatologist [14,15]. Several studies have shown that screening and specific access structures to rheumatology care are effective in reducing the time between disease onset and specialist contact [3,16,17,18,19,20,21]. This was ultimately prompted by the far too high rate of non-inflammatory patients among initial presentations to rheumatology departments. In our study, 74.3% of patients referred for evaluation did not have IRD. Fittingly, a Canadian study reported only 344 patients with confirmed inflammatory arthritis among 3476 referred initial presentations [22]. In an evaluation of 2430 patients, Widdifield et al. [23] found only 31% of patients with an inflammatory diagnosis. Based on the catalogue of entry criteria for EAC appointments, the proportion of confirmed inflammatory diagnoses in Keysser et al. [24] was increased to 40.3%, which is slightly higher than the 32.9% of inflammatory diagnoses in our EAC based on telephone interviews. Interestingly, the median duration of symptoms before presentation to a rheumatologist in the EAC was 6 months, compared with 12 months in the comparison group that did not meet the EAC criteria in the study by Keysser et al. [24]. In addition, the variance was much greater in the comparison group than in the EAC. This supports our hypothesis of a relatively well-defined onset of symptoms in IRDs, as well as the 6-month time interval we chose. Fuchs et al. [25] also investigated the time between symptom onset and rheumatology consultation in patients presenting for the first time at a rheumatology outpatient clinic. The waiting time was significantly shorter in patients with a confirmed inflammatory diagnosis than in non-inflammatory patients: 26 vs. 35 weeks (*p* = 0.007). Interestingly, the waiting time of 26 weeks in patients with IRD was again within the 6-month window we chose. In the study by Thoms et al. [26], an increasing duration of complaints (in this case arthralgia) was also identified as a negative predictor for the presence of IRD. It is this aspect of commonly defined symptom onset specific to IRDs that our prioritisation approach exploits and appears to be able to distinguish well from non-inflammatory disease patterns. In particular, given the high NPV (categorisation into elective consultation) of 80%, we succeed in identifying the non-inflammatory cases. The PPV after categorisation into the early arthritis clinic group was slightly lower at 69%. This raises the question of whether requesting an urgent appointment for an initial presentation by the referring physician is likely to increase the rate of IRDs. However, even in the case of an “urgent” presentation (emergency zone) by the primary care physician, the rate of non-inflammatory diagnoses in our collective was 54.1%. This figure seems to be high, suggesting that a better assessment of the likelihood of an IRD is needed [27].

Interestingly, regardless of the type of early arthritis clinic concept applied, based on a telephone interview, as in our case, a run-in consultation, or a review of previously submitted findings by the rheumatologist, the proportion of IRDs in the presented patients seems to be increasable to a maximum of about 50%, based on the available literature [7]. From the authors’ point of view, it should be considered that screening itself does not lead to a disproportionate consumption of rheumatologic resources, which would then no longer be available for the care of patients with inflammatory rheumatic diseases. In this regard, the authors believe that an effective, ideally delegable, approach is needed that also preserves the resources of referring physicians. Furthermore, it is quite conceivable that in the future AI-based concepts or online portals for patients will allow further triage or prioritisation [28,29]. However, the available data are not yet convincing. For example, based on the online-based symptom checker RhePORT for patients, only a sensitivity of 53.7% and a specificity of 51.8% for IRDs could be found [30]. Proft et al. [31] compared an online-based symptom checker (OSR) for axial spondyloarthritis with physician referral for evaluation of axial spondyloarthritis. A total of 35 patients (19.4%) in the self-referral (OSR) group and 71 patients (39.2%) in the physician referral group were diagnosed with axial spondyloarthritis.

The diagnostic spectrum in the work of Keyser et al. was comparable to that in our collective. The most common diagnosis in the EAC was rheumatoid arthritis (23.8%), followed by peripheral predominant arthritis (5.2%), polymyalgia rheumatica (4.8%), axial spondylarthritis (4.4%), other arthritides (4.8%), and connective tissue diseases (1.2%). A similar distribution of diagnoses was also found by Voigt et al. [5] based on an open run-in consultation. In this study, the duration between symptom onset and presentation at an open consultation was 6 weeks for polymyalgia rheumatica and 4 weeks each for Lofgren’s syndrome and arthritis urica. In our study, polymyalgia rheumatica also dominated in the emergency zone because of the exclusive access provided by the physician contact with the shortest waiting time. This may be due to the usually high burden of complaints. On the other hand, 13.8% of patients with a low pretest probability of IRD were still diagnosed with IRD based on our telephone interview. A major limitation of the work is that patients who were not diagnosed with IRD at the first visit were not followed up for a second assessment. It cannot be completely excluded that IRD may have been diagnosed over time in some of these patients.

The addition of simple serologic markers of inflammation, such as CRP or ESR in screening, increases the overall predictive probability for the presence of IRD, as shown in our work. However, the authors are aware that this effect may be less pronounced or even detrimental in diseases such as connective tissue diseases or spondyloarthritis. The inclusion of CRP and ESR in these cases would reduce the sensitivity for connective tissue diseases or spondyloarthritis. On the other hand, elevated levels of CRP are found in obesity, for example, regardless of the presence of IRD. Interestingly, in the study by Keysser et al. [24] inflammatory parameters (elevated CRP, accelerated ESR, or both) were found not only in the early arthritis clinic (51%) but also in the comparison group (38%).

As expected, the proportion of patients initiating therapy with glucocorticoids or DMARDs was very low in the elective appointment category, at 9.2% and 8.6%, respectively. Significantly more therapy initiations were found in the appointment categories EAC and emergency zone. Especially in the latter, with a comparable proportion of patients starting DMARD therapy (29.7% emergency zone vs. 25.3% EAC), the high proportion of glucocorticoids (44.3% emergency zone vs. 26.2% EAC) may be due to the high proportion of patients with polymyalgia rheumatica. Assigning an appointment request based on the telephone interview to an EAC or emergency zone appointment type resulted in a significantly higher rate of DMARD initiation. This result underlines the potential of the approach presented here with regard to the goal of early therapy initiation. A substantial number of patients did not receive prednisolone or DMARDs despite having an inflammatory diagnosis. This includes the group “arthritis, other than RA or pSPA”. These patients, despite having an inflammatory origin, were treated according to their specific diagnosis, e.g., with colchicine or NSAIDs, rather than with prednisolone or DMARDs. These patients also benefit from early diagnosis and treatment, particularly in terms of rapid symptom control.

The main limitations of the study are its retrospective and single-centre design. In the future, it would be desirable to evaluate the presented approach in a multicentre and prospective setting. Another limitation is that patients without an IRD (“non-inflammatory”) at the initial assessment were routinely seen only once and not systematically followed up. Therefore, it is possible that some patients were subsequently diagnosed with an IRD. However, the authors estimate this would likely involve a very small percentage, as any new symptoms indicative of an IRD would have prompted a return visit to the authors’ centre, e.g., initiated by the primary care physician.

The present study confirms the high proportion of non-inflammatory complaints among initial presentations in routine rheumatology. This contributes significantly to the scarcity of available appointments and thus to delays in diagnosis and initiation of therapy in patients with IRD. The method of algorithm-based patient allocation presented in this paper is an efficient and delegable method that conserves physician resources. It succeeds in prioritising assigned appointments based on symptom duration and significantly increases the proportion of patients with IRD and initiation of specific therapy in the EAC.

## 5. Conclusions

- The present study confirms the high proportion of patients with non-inflammatory complaints at initial presentation, consistent with the literature;
- A duration from symptom onset to appointment request of ≤6 months increases the likelihood of diagnosing an inflammatory rheumatic disease;
- DMARDs were initiated significantly more often in the early arthritis clinic or at emergency visits;
- The algorithm-based allocation approach presented saves time and personal resources, and therefore appears practical for routine care use.

## Figures and Tables

**Table 1:** Patient characteristics in the total study sample (C-reactive protein [CRP], erythrocyte sedimentation rate [ESR]).

	Patients with Inflammatory Disease (n = 361)	Patients with Non-Inflammatory Disease (n = 1046)	*p* Value
Age (years)	60.89 ± 17.2	51.91 ± 15.5	*p* < 0.001
Gender			
Female sex (%)	181 (51.1%)	738 (70.6%)	
Male sex (%)	180 (49.9%)	308 (29.4%)	*p* < 0.001
CRP (mg/dL)	1.71 ± 2.67	0.37 ± 0.69	*p* < 0.001
ESR (mm)	25.28 ± 23.14	10.26 ± 9.60	*p* < 0.001
Triage:			
Elective appointment	91 (13.8%)	569 (86.2%)	
Early arthritis clinic (EAC)	185 (32.9%)	377 (67.1%)	
Emergency appointment	85 (45.9%)	100 (54.1%)	*p* < 0.001

## Data Availability

The data underlying this article will be shared on reasonable request to the corresponding author.
